# The Compressive Behavior and Crashworthiness of Cork: A Review

**DOI:** 10.3390/polym14010134

**Published:** 2021-12-30

**Authors:** Claudia Sergi, Fabrizio Sarasini, Jacopo Tirillò

**Affiliations:** Department of Chemical Engineering Materials Environment, Sapienza Università di Roma and UdR INSTM, 00184 Rome, Italy; fabrizio.sarasini@uniroma1.it (F.S.); jacopo.tirillo@uniroma1.it (J.T.)

**Keywords:** cork, agglomerated cork, compressive behavior, strain rate, anisotropy, temperature, impact, crashworthiness

## Abstract

Cork, a natural material from renewable resources, is currently attracting increasing interest in different industrial fields because of its cellular structure and the presence of the flexible suberin as its main chemical component. In an agglomerated form, it proved to be a compelling product not only as a thermal and acoustic insulator, but also as core material in sandwich structures and as a liner or padding in energy absorbing equipment. From this perspective, the assessment of its compressive response is fundamental to ensure the right out-of-plane stiffness required to a core material and the proper crashworthiness in the safety devices. Considering the complex nature of cork and the resulting peculiar compressive response, the present review article provides an overview of this paramount property, assessing the main parameters (anisotropy, temperature, strain rate, etc.) and the peculiar features (near-zero Poisson’s ratio and unique dimensional recovery) that characterize it in its natural state. Furthermore, considering its massive exploitation in the agglomerated form, the design parameters that allow its compressive behavior to be tailored and the operating parameters that can affect its crashworthiness were assessed, reporting some potential industrial applications.

## 1. Introduction

Waste generation is the result of population growth and industrial development, while waste disposal is a serious issue that pertains to all countries all over the world. According to the data provided by What a Waste 2.0 [1], waste increased from 1.3 billion tons in 2012 to 2.01 billion tons in 2016, and the forecasts for 2030 and 2050 suggest a further increase to 2.59 and 3.40 billion tons, respectively. Of all these waste materials (44% food, 17% paper, 12% plastic, 5% glass, 4% metal, etc.), only a scant amount is virtuously disposed through composting (5.5%) or restored through recycling (13.5%) at the end of its life cycle, whereas the great majority is sent to landfills (controlled, 4%; sanitary, 7.7%; unspecified, 25%), open dumps (33%) and incineration (11%). To counteract the environmental pollution, many countries promulgated new guidelines and regulations such as the ones approved in Europe for waste management (2008/98/EC), packaging and packaging waste (94/62/EC), landfills (1999/31/EC) and end of life of vehicles (2000/53/EC). A suitable way to face the waste disposal issue is to replace the common synthetic non-biodegradable materials with bio-based ones that are from renewable resources and, in most cases, are also biodegradable.

A natural cellular material that has aroused great interest in many different industrial fields is cork, i.e., the bark of the *Quercus suber* L. oak. This evergreen tree grows mainly in west Mediterranean regions in the south of Europe, such as Portugal, Italy and Spain, and in the north of Africa, where the climatic conditions are optimal [2,3], and thanks to its unique features, it is proven to be a versatile material. Flexibility, resilience, chemical stability and flame retardancy are only some of the characteristics that make cork an appealing material. Interested readers can refer to the following reviews and books [2,3,4,5,6]. Indeed, its lightness ensures its feasibility for floating products, the peerless dimensional recovery capacities make it the first choice for energy absorbing devices and this characteristic, together with a low permeability to liquid and gases, provides a good solution for sealing products. Moreover, the excellent electric, sound and thermal insulating capabilities and the good damping properties promote its application for insulation purposes and the good wear resistance and anti-sliding properties ensure its applicability for floor covering and paneling [3,7,8,9]. Some reference values for all these properties that characterize cork are summarized in Table 1.

Natural cork planks are mainly exploited for the production of stoppers [2,9], but over time other cork products were developed to take advantage of stoppers by-products that otherwise would have been squandered. Agglomerated cork, expanded cork agglomerates and cork polymer composites, i.e., rubber-cork [18,19], are the main outcomes of this optimization process. Agglomerated cork is obtained by mixing together cork granules with a thermosetting or a thermoplastic resin and subjecting the plank to a moderate pressure and heating to enhance polymer curing for thermosetting and polymer softening for thermoplastics to achieve a good bonding. Expanded cork is a fully natural product where granules are bonded together through the chemical compounds resulting from the partial chemical degradation of cork components as a consequence of superheated steam exposure. Finally, rubber-cork is obtained by cross-binding cork granules with synthetic or natural rubbers to achieve the best combination of both materials properties [3].

All these types of cork are characterized by different mechanical properties that enable their application in numerous fields such as insulating boards in the case of expanded cork and core material in sandwich structures or energy absorbing devices in the case of agglomerated cork. Despite the pronounced differences in performance, all cork types are characterized by a common feature, namely a peculiar compressive response derived from cork’s microstructure and chemical composition, which provide them with an excellent resilience, good energy absorption capacities and, above all, an outstanding dimensional recovery. In light of these peculiarities, many studies assessed the compressive behavior of the different cork products evaluating the effect of numerous design and operating parameters, e.g., strain rate, temperature, anisotropy, granules dimension, binder type and amount, etc. The results obtained increased the awareness of the crashworthiness of this material endorsing further studies to ensure its exploitation in safety and energy absorbing devices. Considering the vast state of the art concerning cork’s compressive response and its crashworthiness, the present work aims to provide a detailed and thorough review on this topic for both natural cork and agglomerated cork.

## 2. Natural Cork

**Figure 1:**
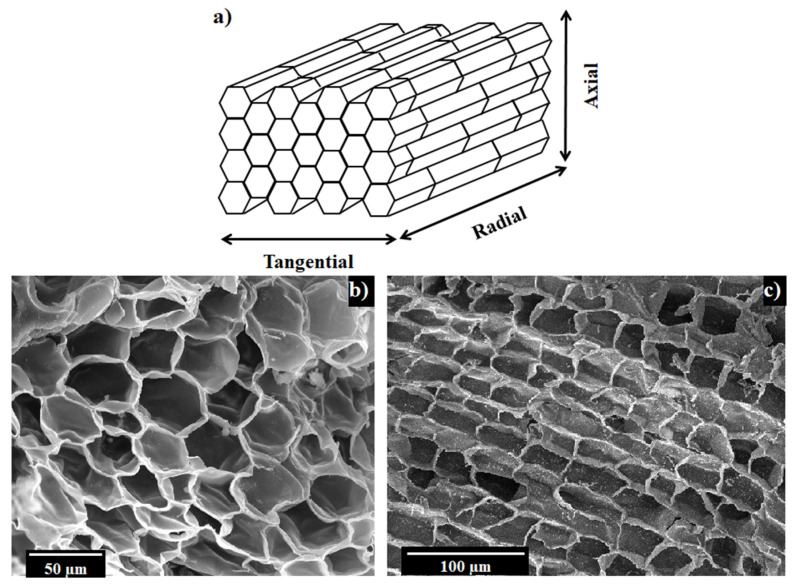
Schematic reconstruction of cork’s cellular microstructure (**a**), cork micrograph perpendicular to the radial direction (**b**), cork micrograph perpendicular to the tangential/axial direction (**c**).

Cork is a cellular material with a honeycomb-like microstructure where prismatic closed cells, with a polygonal base ranging from four to nine edges (Figure 1b), are stacked base-to-base along the radial direction of the tree as schematically shown in Figure 1a [2]. Cork cells are filled with an air-like gas mixture and, when observed perpendicularly to the tangential/axial direction, display a brick-layered 2D arrangement (Figure 1c). Despite the overall uniformity and regularity of cork’s microstructure, a certain heterogeneity is brought by the lenticular channels, which are pores that radially cross cork bark to ensure gas exchange between the external environment and the inner tissue of the plant and are characterized by a 0.1–100 mm^2^ cross section [8]. Another feature that characterizes cork’s microstructure is the corrugation of the lateral walls of the prism, which displays, normally, two undulations per face [8]. All these characteristics make its cells behave as auxetic “pads” [20] and are responsible for the peculiar compressive response of cork that was first studied and acknowledged by Gibson et al. [9] in 1981.

**Figure 2:**
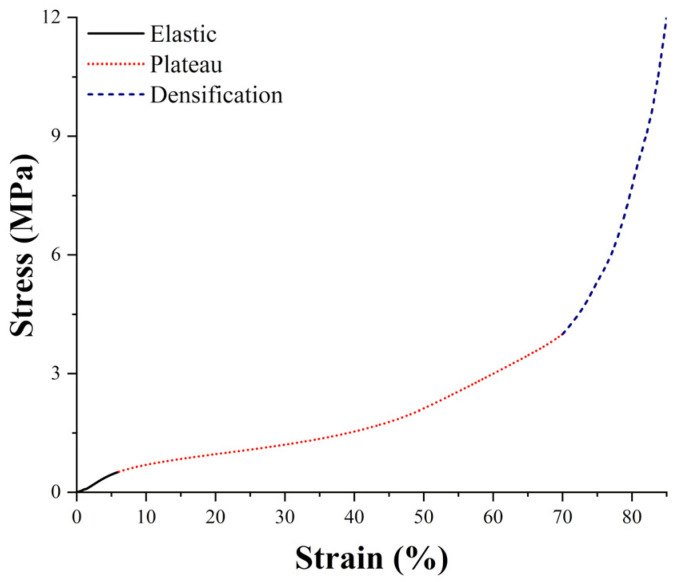
Typical stress–strain compressive curve of cork.

Cork’s compressive curve is perfectly comparable with cellular foam ones and is characterized by three main regions, as shown in Figure 2. The first region is the linear elastic region that extends up to 5–7% strain, where cell walls’ elastic collapse occurs, determining the beginning of the second section, i.e., the plateau region. This portion of the curve extends up to almost 70% strain, depending on the cork’s density, where complete cell collapse occurs, marking the transition to the densification region. Here, the interaction of opposite cell walls results in a steep increase in the stress for small deformation increments [8,9]. The plateau region strain limit, and therefore the densification strain value, decrease for increasing cork densities because the higher compactness of the material tends to trigger opposite cell walls’ mutual contact. This behavior was modelled by Saadallah [21], who employed at first a crude trilinear model where each region was represented by a line with a different slope and at a later time moved to a more accurate nonlinear model with a third order polynomial regression, which allowed for the improvement of the model’s accuracy and the reduction in the number of parameters needed.

The greatest merit of cork lies in the ability of its cell walls to bend and fold completely, even at high strains, without experiencing fracture or damage. This capability can be ascribed to cell walls corrugations that act as preferential folding paths, but also to the remarkable flexibility of suberin, which is cork’s main structural chemical component (around 53%) [8]. Suberin is a glyceridic polyester where the linear long chains of fatty acids and alcohols are assembled in a ribbon-like structure. The other main chemical components of cork are lignin, cellulose and hemicellulose and their average content is summarized in Table 2, which also includes the non-structural components, i.e., extractives and others. Once the key features that characterize cork’s compressive behavior have been pointed out, it is possible to proceed with a more accurate analysis of all the other parameters that influence its response.

### 2.1. Anisotropy

As pointed out in the previous section, natural cork is characterized by an intrinsic anisotropy in the microstructure, which leads to an anisotropic compressive response, as reported by Gibson et al. [9], by Pereira et al. [24], by Anjos et al. [25,26] and by Oliveira et al. [27]. This anisotropy does not affect the typical shape of cork’s compressive curve, but it is responsible for a variation in its typical compressive properties, i.e., compressive modulus, plateau stress, densification stress and strain. Neglecting the other parameters assessed in each study and focusing only on the differences due to testing direction, all authors report superior compressive properties, in terms of compressive modulus and plateau stress at 5% strain, along the radial direction with respect to both the axial and tangential directions. In particular, a higher compressive modulus between 4.7 and 66.07% and a plateau stress between 3.4 and 131% were observed in the radial direction, as it can be inferred from the data reported in Table 3. If all authors agree that the radial direction is characterized by a compressive response superior to the tangential and axial ones, some discrepancies can be observed when comparing the tangential and axial directions. In particular, the data reported by Pereira et al. [24] and by Anjos et al. [25] disclose the better performance of the axial direction with respect to the tangential one, whereas Anjos et al. [26] display the superior mechanical properties of the tangential direction with respect to the axial one, especially considering the plateau stress values. The disagreement can be solved considering the results provided by Gibson et al. [9] and by Oliveira et al. [27], who found almost equal results along the above-mentioned directions and ascribed the differences observed in the other studies to the natural origin of cork, which is responsible for the extreme variability of the mechanical properties, as already observed for vegetable fibers [28]. In conclusion, cork can be considered almost symmetrical along the radial direction and hence isotropic in the plane normal to the radial direction.

### 2.2. Other Parameters Affecting Cork Microstructure: Density, Porosity, Growth Rate and Origin

In addition to the intrinsic microstructure anisotropy, there are many other parameters that affect natural cork’s microstructure and hence its compressive behavior, such as density and porosity [25,26], growth rate [24] and origin [27]. Concerning density’s effect, two works by Anjos et al. [25,26] revealed an increase in both compressive modulus and plateau stress for an increasing cork density, especially along the radial direction, and a reduction in the densification strain entailing an anticipation of the densification process. These results are strictly correlated to cork’s microstructure, i.e., cell walls’ thickness and undulation, but also to the macro-porosity resulting from the lenticular channels that cross the cork planks along the radial direction to ensure gas exchange between the tree and the external environment [8]. If density and porosity are strictly correlated in most materials and usually the higher the density is, the lower the porosity is, this correlation cannot be directly assumed for cork as proven in a study by Anjos et al. [25], who reported a complete lack of correlation between the two parameters. A further verification was provided in [26], where the denser cork displays a higher porosity, i.e., 9.4% against 5.1%, and a higher number of pores, i.e., 11 against 7. In this case, the higher porosity is counteracted by the presence of lignified schlerenchymatic cells that develop around the lenticular channels and are responsible for the cork’s density increase being characterized by thicker cell walls than cork ones. The lignocellulosic nature of these cells makes them more resistant, thus providing the cork with higher compressive properties, despite the higher porosity.

Growth rate and cork origin are two other factors that significantly influence cork’s microstructure and hence the resulting compressive properties. Pereira et al. [24] investigated 9-year aged cork planks grown with a different rate and hence characterized by a different caliber. They found out that the higher the tree rings’ growth rate is, hence the caliber of the natural cork, the lower the compression strength and modulus are. The authors ascribed this trend to the higher corrugation observed in the early cork cell walls of the small caliber cork, which, in extreme cases, can even lead to their collapse on the late-cork cells that constrained their growth. The authors’ hypothesis can be corroborated by a clear decrease in the densification strain of thin caliber cork with respect to large caliber cork, suggesting a real pre-collapse of its cell walls, which triggers the densification phenomena. Cork’s growth rate is influenced by several environmental variables, such as rainfall, temperature and type of soil, meaning that the growth place and hence the cork’s origin play a significant effect on its microstructure, as proven by Oliveira et al. [27], who assessed the variability of location growth on cork’s properties considering 10 different sampling sites. They found out that the geographical location of cork production is a significant factor of variation in cork’s compressive properties determining, for example, a variation between 9.1 and 12.3 MPa of the compressive modulus along the radial direction.

These results underline one of the main weaknesses of natural materials over synthetic ones, namely, the variability of their mechanical properties with respect to the more standardized ones achievable through a controlled manufacturing process.

### 2.3. Effect of Testing Parameters and Conditions: Strain Rate, Stress Relaxation and Creep

If cork’s microstructure is the primary factor influencing natural cork behavior, it must be considered that even testing parameters and conditions can influence and modify its response. Rosa and Fortes [29] were the first to consider the strain rate sensitivity of cork and to disclose its viscoelastic nature with increasing strain rates, i.e., 2.1 × 10^−4^, 2.1 × 10^−3^ and 2.1 × 10^−2^ s^−1^, through quasi-static compression tests. The viscous component was investigated in the 10^−4^−10^−2^ 1/s range and was proven to play a significant role on cork’s compressive response, determining an increase of 116.6% and 22.1% in the compressive modulus and plateau stress at a 10% strain along the radial direction, of 93.5% and 42.85% along the axial direction and of 155% and 57.4% along the tangential direction. Cork’s strain rate sensitivity was also evaluated and a fairly isotropic mean value of 0.06 was identified. This invariability along the three main directions is consistent with the fact that material viscoelasticity and strain rate sensitivity are mainly related to its chemical composition and to its macromolecules length rather than its microstructure.

Rosa and Fortes [30] also investigated the stress relaxation and creep behavior in the compression of cork. Concerning stress relaxation, a linear evolution of the logarithm of the stress normalized in respect to the applied deformation with the logarithm of time was identified. The slope of the curves proved to be almost constant irrespective of the selected strain and the testing direction, determining the isotropic response of cork. A certain effect of density was observed with an increase in the curve slope from 0.045–0.06 for the denser cork to 0.065–0.075 for the lighter cork. Concerning the creep response, the authors applied different constant stresses in order to work in different regions of the compressive curves and to assess the effect of the different deformation mechanisms, i.e., walls bending in the elastic region, walls buckling in the plateau region and walls crushing in the densification region. The strain rate in the initial part of creep curves proved to increase when the applied load increased and a significant variation in its value was observed depending on the test direction, thus highlighting again the anisotropic nature of cork. They also pointed out how the wall crushing mechanism is a slower process than walls buckling.

### 2.4. Working Environment

Anisotropy, density and strain rate sensitivity are all parameters that influence cork’s compressive properties and need to be considered when designing a component with this natural material, but another determinant that cannot be neglected because it can significantly modify cork’s microstructure and chemical composition and hence the resulting properties, is the environment in which it is meant to be treated or employed. In particular, temperature and moisture are two key factors that can significantly deteriorate cork’s mechanical properties, jeopardizing its suitability for a specific application.

**Figure 3:**
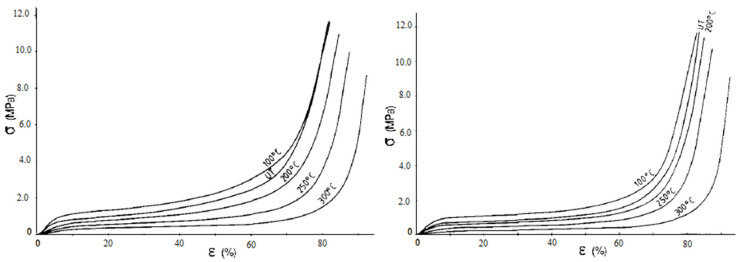
Compressive curves along the radial and axial directions of untreated (UT) and 1 hour thermally treated corks (Reprinted with permission from [32]).

Despite promising fire-retardant capabilities, the exposure of cork to high temperatures can significantly modify its chemical composition, as proven by Pereira [31], who investigated the thermochemical degradation of cork and identified 150 °C as the temperature threshold at which thermal decomposition in air begins. Polysaccharides and extractives proved to be the most thermosensitive components, hemicellulose completely disappears at 200 °C and cellulose is considerably degraded at this temperature, whereas the more thermally stable suberin only significantly decomposed above 250 °C. These changes in cork’s chemical composition inevitably induce changes in cork’s mechanical properties as foreseen by Rosa and Fortes [32] and Rosa and Pereira [33], who investigated the alterations induced by temperature on the compressive response of cork for a short time (15, 30 and 60 min) in the range of 100–300 °C and for a longer time (1, 7, 28 and 42 days) in the range of 100–150 °C, respectively. Figure 3 is reproduced from Rosa and Fortes’ study [32] and shows the compressive curves along the radial and axial directions of untreated and thermally treated corks.

A clear decrease in the compressive properties of cork can be observed at 200, 250 and 300 °C, whereas an increase in the plateau stress can be observed at 100 °C. In particular, the exposure at 300 °C causes a reduction of 15 times in the Young’s modulus and the exposure at 200 °C a decrease of three times, whereas the slight increase in the compressive properties experienced at 100 °C may be ascribed to the water and moisture removal. The results are, therefore, in perfect agreement with the 150 °C threshold limit that was reported by Pereira [31] and point out the strict correlation between chemical composition and cork performance. Once the safe temperature working range has been ascertained, evaluating the exposure time effect becomes relevant and, in this framework, the work proposed by Rosa and Pereira [33] allows us to understand the combined effect of time and temperature. According to their study, Rosa and Fortes pointed out a change in the mass and chemical composition of cork, but the degradation mainly concerns polysaccharides and extractives, which are not the structural constituents of the material, thus determining only a slight decrease in the compressive properties after 42 days at 100 °C. They also report an increase in the aforementioned properties after 42 days at 150 °C, due to the appearance of chemical components deriving from condensation reactions between thermal degradation products, thus proving the feasibility of cork at these temperatures, even for prolonged exposure periods.

As previously mentioned, even moisture influences cork’s compressive behavior as proved by Lagorce-Tachon et al. [34], who assessed the effect of relative humidity (RH) on cork’s compressive modulus and found out an almost constant behavior up to 53% RH and a consequent decrease due to water molecules clustering, which can act as plasticizer. Both increasing operating temperatures and humidity proved to be harmful to cork’s compressive behavior after a certain threshold, i.e., 150 °C and 53% RH, but their combined effect can further decrease cork’s tolerance, as proved by Rosa and Fortes [35], who evaluated the effect of water vapor heating at 100 and 300 °C on cork’s compressive response. In their previous study, Rosa and Fortes [32] already pointed out a decrease in cork’s compressive modulus and plateau stress when heated at 300 °C in air, but a further decrease in these parameters was observed when heating in water vapor. However, the detrimental effect of a high temperature and moisture combination is much more evident for specimens tested at 100 °C, which display an increase in both compressive modulus and plateau stress when tested in air but a significant drop when tested in water vapor. A significant outcome is that both the 100 and 300 °C vapor heated samples show a recovery of their properties after drying; in particular, the recovery is almost complete in the case of 100 °C samples, but is lower than air-heated samples at 300 °C. The latter finding allows us to draw the important conclusion that the combined effect of temperature and moisture actually plays a higher detrimental effect.

### 2.5. Peculiar Features: Dimensional Recovery and Poisson’s Ratio

Until now, all the parameters influencing cork’s compressive behavior were considered, but an in-depth analysis is necessary to point out cork’s peculiar features resulting from its compressive tests: Poisson’s effect and dimensional recovery.

Cork is known for being a near-zero Poisson’s ratio material, often considered as a benchmark when trying to design a 3D-printed truss lattice with near-zero Poisson’s ratio [36]. Gibson et al. [9] were the first to estimate cork’s Poisson’s effect and a more accurate analysis was then provided by Fortes and Nougeira [37]. The Poisson’s ratios obtained in each study are summarized in Table 4. Both works considered the two non-radial directions as equivalent, thus assessing three fundamental configurations, i.e., R/NR, NR/R and NR/NR, where the first term identifies the longitudinal direction and the second term the transverse direction. Both works pointed out a certain anisotropy even in cork’s Poisson’s ratio with an almost zero value when the non-radial (NR) and radial (R) direction relationship is considered and a much higher value, between three and four times higher, when the two non-radial directions’ relationship is addressed. Fortes and Nogueira further specified that the reported values were evaluated for low strains, and moving up to 5–8% strains, the Poisson’s ratio becomes almost zero until reaching negative values at larger strains. The extremely low values obtained for R/NR and NR/R are mainly ascribed to the lateral walls’ corrugations of cork that promote a “concertina-like” effect, namely, an ordered folding of the cell walls. Moreover, according to Fortes and Nougeira, if the prism bases of the cells are randomly located with respect to the corrugations, no expansion of the material occurs when the cells are compressed along the radial direction. The differences that arise between Gibson et al.’s and Fortes and Nougeira’s Possion’s values can be ascribed to two main factors. The first one is the higher sample dimensions selected by Fortes and Nogueira, i.e., 30 mm against the 15 mm employed by Gibson et al., which allows the end constraints effect to be minimized. The second factor concerns the use of an apparatus equipped with a transverse extensometer to obtain more accurate measurements along the transverse direction by Fortes and Nougeira. As a conclusion, Fortes and Nougeira’s Possion’s ratio values seem more reliable even if the ones provided by Gibson et al. allow us to draw the same conclusions.

The second peculiar feature that makes cork so attractive as sealant is the outstanding dimensional recovery capability, which was first investigated by Rosa and Fortes [29] and then readdressed by Anjos et al. [26]. In particular, Rosa and Fortes pointed out an almost full recovery of dimensions after low deformations, i.e., ε = 0.3, and a permanent deformation lower than 9% after high deformations, i.e., ε = 0.8. Similar results were reported by Anjos et al., who found out a permanent deformation between 2.9 and 9.3%, depending on the cork’s density and test direction, after a 0.5 deformation. Both studies ascribe this impressive dimensional recovery to the unfolding of the cell walls, which is promoted by relaxation phenomena at the macromolecular level. This unfolding mechanism is mainly due to the flexibility of suberin that prevents cork walls fracturing. Moreover, both works ascribe the permanent deformation resulting from large compressive strains to irreversible fracture phenomena, such as the fracture and collapse of lignified pores with s schlerenchymatic cells. Anjos et al. pointed out how a dimensional recovery is almost instantaneous after unloading, with a recovery of 50% of the deformation during the first day and a recovery of 70% within the second day, but both works are unanimous in defining the dimensional stabilization after 15–20 days from testing. Rosa and Fortes also investigated the effect of strain rate on the cork recovery rate, showing that the higher the strain rate is, the higher the recovery rate is. This could be explained considering that the authors also noticed an increase in the transversal deformation for an increasing strain rate, which may imply a lower degree of order in the cell walls’ folding and hence a higher tendency of the material to move back to its initial state. Anjos et al. assessed the effect of the density disclosing a higher maximum recovery for less dense cork, ascribing this outcome to a lower number of lignified channels that are more prone to fracture and hence to permanent deformation.

## 3. Agglomerated Cork

Natural cork sparked great interest as proven by the numerous works that have assessed its compressive behavior since 1981. Its use as a wine stopper is so well-established that many studies addressed the effect of a cork stopper type on the quality of white [38] and red [39] wines stored in bottles. Thanks to the high standard provided, it maintained its dominance throughout the years and also against synthetic polymeric stoppers, which, however, present some criticalities, such as the dispersion of microplastics in the wine [40]. The massive exploitation of natural cork planks for the production of this primary product led to the formation of huge amounts of by-products. Considering the high potential of this natural material, the byproducts generated from stopper production were suitably redirected to the production of agglomerated cork planks manufactured by mixing cork granules with a polymeric binder and subjecting the blend to pressure and heat. The possibility of tailoring plank properties by selecting the type and amount of polymeric binder, granules dimensions and packing density allows the intrinsic variability of this natural material to be partially overcome, making it an attractive product not only for shoe soles, thermal, acoustic and vibration insulating boards, floor paneling and gaskets, but also as a bio-based core material in the production of eco-friendlier sandwich structures [2,7]. For the latter application, the knowledge of the material’s compressive response is fundamental considering that a good core material must be characterized by a high out-of-plane stiffness to ensure a constant distancing of sandwich skins.

### 3.1. Compressive Behavior

As previously mentioned, the first parameters affecting agglomerated cork’s compressive response are the design parameters such as binder type and concentration, grain size and density, as investigated by Santos et al. [41], Crouvisier-Urion et al. [42] and Jardin et al. [43]. Santos et al. studied three diisocyanate binders with different rigidity (hard, intermediate and flexible), employing three different concentrations (5, 10 and 15%), two different granules size (0.5–1 and 3–4 mm) and three different densities (120, 160 and 200 kg/m^3^). Crouvisier-Urion et al. employed a more rigid aliphatic binder and a more flexible aromatic binder, tailoring their concentration as a function of the two grain size configurations selected, i.e., macro-agglomerated cork (2.5–8 mm) and micro-agglomerated cork (0.5–2.8 mm). Finally, Jardin et al. investigated the agglomerated cork produced with a polyurethane binder and expanded cork produced with its own suberin as a binder assessing the effect of density and granule size. All the works pointed out an increase in compressive stiffness and in the plateau stress employing a more rigid binder, a denser material and a bigger granules size. Concerning the binder concentration, depending on the range investigated, no effect was detected or a partial decrease in the sample stiffness for an increasing binder amount was observed. This must be ascribed to the lower modulus normally possessed by the polymeric binder with respect to cork. Santos et al. and Jardin et al. also pointed out the relationship between the decrease in the compressive stiffness and the delay of material densification. This means that if the designer aims to extend the plateau region, it is more feasible to employ a more flexible binder and increase its concentration or reduce its granule size and material density. On the contrary, if the main goal is to increase the level of energy stored per unit volume neglecting the densification stage, opposite choices must be taken. The effects of the discussed parameters on agglomerated cork’s compressive stiffness are summarized in Table 5. Some works tried to further increase design flexibility by evaluating the effect of polymeric binder reinforcement with short fibers [44,45], graphene oxide (GO) and nano platelets (GNP) [46]. The two types of reinforcement proved to play an opposite effect. In particular, short fibers determine an increase in agglomerated cork’s compressive modulus and plateau stress, especially along the in-plane directions that experience an increase of 71% in the compressive modulus and of 40% in the plateau stress. On the contrary, the introduction of nanofillers promotes a reduction in agglomerated cork’s plateau stress and a delay in the densification stage. This means that the introduction of fillers in the polymeric binder is another suitable way to tailor agglomerated cork’s compressive properties.

If design parameters are fundamental to tailoring agglomerated cork’s performance, other test conditions can modify agglomerated cork’s response, as already pointed out for natural cork, including sample orientation [47,48], strain rate [47,48,49,50,51,52,53], moisture [42], temperature [49,50] and number of loading cycles [47,54].

**Figure 4:**
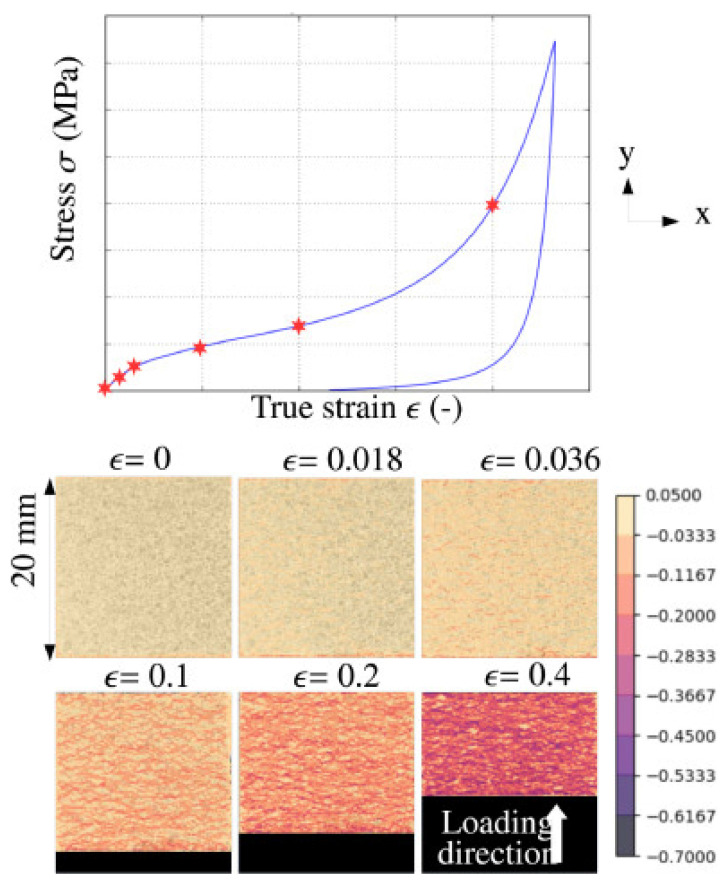
Longitudinal strain map at different moments of agglomerated cork compression (Reprinted with permission from [47]).

The first outcome to underline is agglomerated cork’s anisotropy; in fact, even if the random orientation of cork granules should ensure almost isotropic mechanical properties to counteract the anisotropic nature of natural cork, agglomerated cork is characterized by an in-plane stiffness higher than an out-of-plane one. This must be ascribed to the production process that promotes agglomeration and polymeric binder curing through the application of moderate pressure and heating. The applied pressure tends to induce residual stresses in the material [3] and this seems to be confirmed by the results reported by Sergi et al. [48], who found out a higher discrepancy between the in-plane and out-of-plane properties of the denser cork, which experiences higher residual stress due to its higher compactness whereas the less dense cork seems nearly isotropic. Agglomerated cork can be assimilated to a multiscale foam and the random orientation of the granules intended to obtain an almost isotropic material at the macro level is actually responsible for material anisotropy at the micro level. Through DIC analysis, Gomez et al. [52] disclosed a severe heterogeneity in the specimens responsible for a strong difference in the strain of different points in the sample and similar results were reported by Le Barbenchon et al. [47] and by Sasso et al. [53], who identified several localization bands with different regions experiencing different strains, as reported in Figure 4.

Moving to strain rate sensitivity, many studies investigated the effect of test speed on agglomerated cork exploring the quasi-static regime [47,49,50], i.e., 10^−5^–10^−1^ s^−1^, the intermediate range [52], i.e., 25–75 s^−1^, the low-high range [51,53] i.e., 10^−3^ and 100–600 s^−1^, and the whole strain rate range [48], i.e., 10^−3^–10^−1^ and 60–200 s^−1^. All studies report an increase in agglomerated cork’s compressive modulus, plateau stress and densification stress for increasing the strain rate confirming the viscoelastic nature already acknowledged for natural cork. This characteristic is not affected by the agglomeration process considering that the binder is also a polymer. The investigation of the low strain rate range pointed out a linear dependency of compressive properties with strain rate, but the assessment of the whole strain rate range disclosed a change in agglomerated cork’s behavior moving from the low-medium regime to the medium-high regime. In particular, a change of one order of magnitude was observed moving from the first to the second region with a strain rate sensitivity between 0.029 and 0.041 [48] in the low-medium strain rate range, which is perfectly comparable with the 0.06 one reported by Rosa and Fortes for natural cork in quasi-static conditions [29]. The small differences between the strain rate sensitivity of these two types of cork can be likely ascribed to the presence of the polymeric binder. Strain rate proved to play a strong effect even on the two main peculiar features of agglomerated cork, i.e., the Poisson’s ratio and the dimensional recovery. Gomez et al. [52] showed an increase of 33% in a 140 kg/m^3^ agglomerated cork’s Poisson’s ratio moving from the quasi-static to the high velocity regime. This study also pointed out a progressive decrease in this parameter up to 12% strain advising to include Poisson’s ratio dependency on both the strain and strain rate in a numerical model to obtain a reliable analysis of this natural material. Sergi et al. [49] disclosed a dependency of cork’s instantaneous recovery and permanent deformation on the test speed, highlighting an increase in both parameters with an increasing test speed. Both results are ascribed to the lower deformation time provided to the material, which struggles to comply with the imposed deformation, inducing a higher disorder in cell walls folding. The latter is the driving force for the material to move back to its original state when the load is removed, thus increasing the instantaneous recovery, but it also increases the risk of lenticular channel and pore collapse, which determine a higher residual deformation. These outcomes are compliant with the ones reported by Rosa and Fortes [29] for natural cork, which highlighted an increase in the recovery rate with increasing strain rates.

As already pointed out for natural cork, moisture and temperature are two other factors that can significantly reduce agglomerated cork’s compressive performance. In particular, Crouvisier-Urion et al. [42] found out a significant drop in compressive modulus and plateau stress at 50% strain for a moisture content between 3 and 5 wt%, depending on the polymeric binder content, type and particles size. In particular, an aliphatic polyurethane binder delays the compressive properties drop from a moisture content of 4 to 5 wt% with respect to an aromatic polyurethane binder, while the use of a micro-agglomerated cork with a higher aromatic binder content, i.e., 20% weight/weight cork, anticipates the drop to a 3 wt% moisture content with respect to a macro-agglomerated cork with a lower binder content, i.e., 5% weight/weight cork. Considering the results reported, it can be inferred that particle size plays a more important role with respect to the polymeric binder content. Despite the hydrophobic nature of the binder and its higher content in the micro-agglomerated cork, the latter experiences a decrease in its compressive properties at a lower moisture content. This could be likely ascribed to the higher surface of cork exposed when micro-granules are used. The results proposed by Crouvisier-Urion et al. are coherent with the ones already reported by Lagorce-Tachon [34] for natural cork and must be ascribed to the plasticizing effect played by water, which is much more pronounced in the agglomerated corks with lower amounts of a polymeric binder that is fully hydrophobic. Even rising temperatures from room temperature up to 80 °C proved to be detrimental for the agglomerated cork’s properties, inducing a decrease in both compressive modulus and plateau stress due to the progressive softening of the polymeric binder, which progressively loses its load-bearing capabilities [49,50]. The effect of each aforementioned testing parameter on agglomerated cork’s compressive modulus is briefly summarized in Table 6.

### 3.2. Crashworthiness: Temperature, Multiple-Impacts and Applications

The combination of good energy absorbing capabilities with a unique dimensional recovery endows agglomerated cork with a good crashworthiness and draws attention for safety and energy absorbing devices. In this framework, many research studies decided to delve into agglomerated cork’s dynamic compressive behavior assessing the effect of various design parameters such as specimen thickness [55], operating temperature [48,56,57,58] and number of repeated impacts [41,43,59], also providing thorough comparisons with more traditional synthetic foams [60,61,62] and a numerical simulation of its compressive behavior [58,62,63].

**Figure 5:**
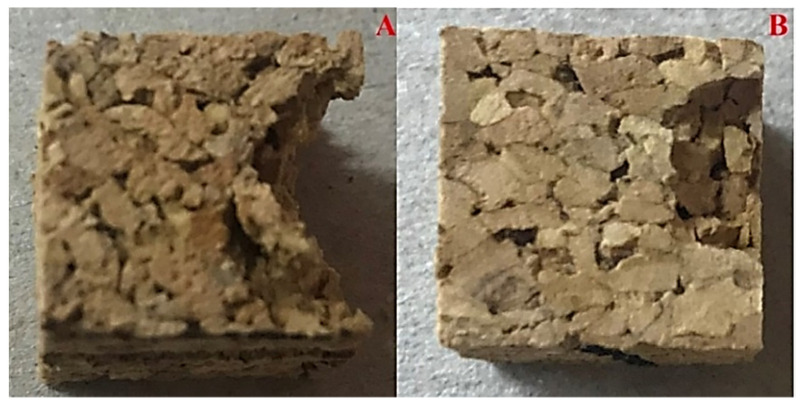
Brittle detachment of cork plugs after performing a dynamic compression at −40 °C on 140 kg/m^3^ (**A**) and 200 kg/m^3^ (**B**) agglomerated corks (Reprinted with permission from [48]).

**Figure 6:**
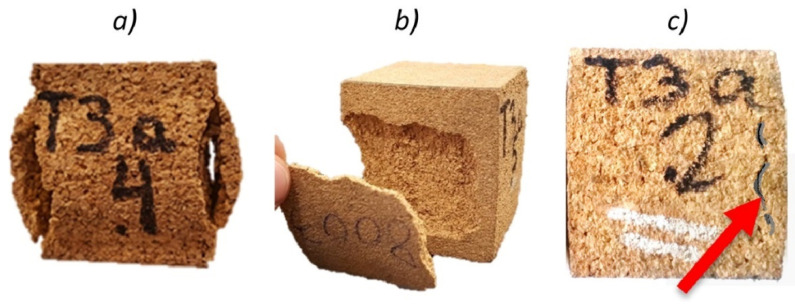
Decohesion of cork plugs after performing a dynamic compression at 100 °C on 168 kg/m^3^ (**a**), 199 kg/m^3^ (**b**) and 216 kg/m^3^ (**c**) agglomerated corks (Reprinted with permission from [57]).

Temperature is the first operating parameter that must be considered because it can significantly affect the crashworthiness of agglomerated cork modifying its energy absorbing reliability. As a general trend, a decrease in the operating temperature from 60/100 °C to −30/−40 °C causes an increase in agglomerated cork’s stiffness with a consequent increase in the plateau stress and a reduction in the maximum displacement, being equal to the impact energy [48,57,58]. This behavior can be mainly ascribed to the glass transition of cork that occurs between 10 and 25 °C [49,64] due to the suberin microcrystalline melting [65] that determines a transition from a strongly viscoelastic behavior to a more glassy one. A further change in the dynamic compressive response of this multiscale foam can be caused by the thermo-mechanical modification experienced by the polymeric binder. For example, the approach of the binder glass transition temperature, e.g., −40 °C for the polyurethane binder [49,64], can promote an extremely brittle response of the specimen with the consequent detachment of cork plug, as proven by Sergi et al. [48] after an impact at −40 °C (Figure 5). Even the use of high temperatures can have a detrimental effect on the polymeric binder, causing an excessive softening that can compromise its adhesive performance, as proven by Kaczynsky et al. [57] with a 100 °C impact (Figure 6).

Once all the critical temperature thresholds are identified, a more thorough understanding of cork’s energy absorbing capabilities can be achieved. Being equal to the impact energy and selecting a fixed strain value, i.e., 63%, Kaczynsky et al. [57] found out a decrease in the energy absorbed by agglomerated cork with the temperature rising from −30 to 100 °C, (−30 °C, −15 °C, 0 °C, room temperature and 100 °C). This must be ascribed to the fact that for a fixed strain, the lower the temperature is, the higher the plateau stress is and hence the energy absorbed by the material. This causes a reduction in this parameter to less than a quarter moving from −30 to 100 °C. It must be pointed out that the reduction in the energy absorbed moving from −30 °C to room temperature is much more severe than the one experienced moving from room temperature to 100 °C. These results can be ascribed to the glass transition of cork, which occurs around room temperature, as already pointed out. This hypothesis is further corroborated by the results provided by Ptak et al. [58], who found comparable energy absorbing capabilities for agglomerated cork tested at 21 and 50 °C, employing the same impact energy and the same displacement cut-off. This low variability in the energy absorption capabilities above room temperature was also acknowledged by Sergi et al. [48], who found a comparable percentage absorbed energy moving from room temperature to 60 °C. The main differences between the work proposed by Sergi et al. and by Kaczynsky et al. arise in the energy absorbing capabilities moving from very low temperatures, i.e., −40 °C and −30 °C, respectively, to room temperature. In particular, with equal impact energy, Kaczynsky et al. disclosed a decrease in the absorbed energy for an increasing temperature as a consequence of the cut-off determination and plateau stress increase, whereas Sergi et al. found a higher percentage of absorbed energy for increasing operating temperatures, accounting for the overall impact response and hence considering the higher deformability due to the viscoelasticity of cork at higher temperatures.

Another key design parameter is the number of impacts that the natural multi-scale foam is expected to withstand. The multiple-impacts performance of agglomerated cork was investigated by Santos et al. [41], by Jardin et al. [43] and by Sanchez-Saez et al. [59] and was further analyzed by research studies that provided a thorough comparison with traditional synthetic foams such as EPS (expanded polystyrene) [61], EPP (expanded polypropylene) [62] and PVC (polyvinyl chloride) [60]. Sanchez-Saez et al. [59] investigated the three-impact response of 140 kg/m^3^ agglomerated cork, assessing three different thicknesses and two different impact energies. They found an increase between 13 and 21% in the maximum strain, an almost constant maximum force and a slight reduction between 3 and 6% in the percentage absorbed energy moving from the first to the third impact when cork was tested within its plateau region. This confirms its excellent energy absorbing capacities throughout subsequent impacts. Similar results were reported by Jardin et al. [43] for three agglomerated cork densities, i.e., 178, 199, 216 kg/m^3^, which experienced an increase between 2 and 35% in impactor acceleration moving from the first to the second impact. A deeper analysis was provided by Santos et al. [41], who investigated the effects of binder type, binder concentration, sample density and granule size in order to tailor agglomerated cork’s multiple-impacts response. The results obtained for two consecutive impacts proved that (i) a less flexible binder allows the impactor acceleration to reduce and the absorbed energy at the second impact to increase slightly; (ii) a lower binder concentration allows the energy absorbed at the second impact to remain high but does not have an effect on impactor acceleration, contrary to the binder type; (iii) an increase in sample density from 160 to 200 kg/m^3^ allows the impactor acceleration at the second impact to reduce by almost 30% with no significant differences in the absorbed energy; (iv) larger granules size reduces the impactor acceleration at the second impact by almost 10% with no significant differences in the absorbed energy.

**Figure 7:**
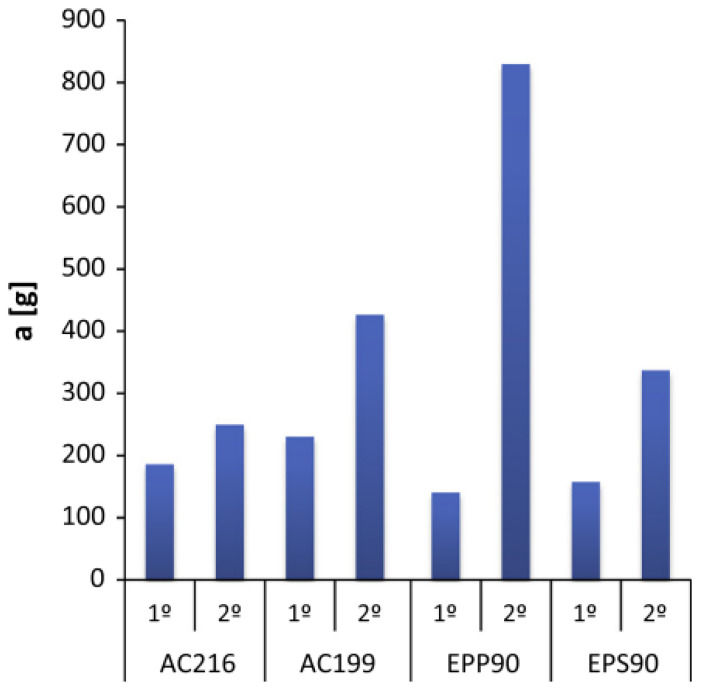
Impactor acceleration after the first and second impact for two agglomerated corks (AC) with 216 and 199 kg/m^3^ density and EPP and EPS foams with 90 kg/m^3^ density (Reprinted and adapted with permission from [62]).

In light of this design freedom, which allows the agglomerated cork’s crashworthiness to be tailored as a function of the main design parameters, the feasibility of agglomerated cork in safety and energy absorbing devices was further investigated, providing a suitable comparison with well-established synthetic foams. In their comparative work, Miralbes et al. [61] pointed out that agglomerated cork and EPS foam display a similar average deceleration of the impactor, i.e., 389.7 m/s^2^ for agglomerated cork and 372.1–416.8 m/s^2^ for EPS foams, but agglomerated cork is able to provide a much lower deceleration peak of 1475.6 m/s^2^ compared to the 1508.8–2078.9 m/s^2^ of the EPS foams. Even from the Head Injury Criterion (HIC) and dimensional recovery point of view, agglomerated cork performs better than EPS, providing an HIC equal to 385, which is significantly lower than the 633–660 observed for the EPS foams, and a dimensional recovery of 82.7%, which is significantly higher than 28.57–36.5% of the EPS foams. Similar results were reported by Fernandes et al. [62], who, in a two-impact study, reported a much lower acceleration after the second impact for agglomerated cork with respect to EPP (expanded polypropylene), as shown in Figure 7. The two studies prove the feasibility of agglomerated cork as a liner for helmets with an improved eco-sustainability and biodegradability, but they also point out that cork products are characterized by a higher density, hence the specific energy they are able to absorb is considerably lower and they can be suitably employed as a liner only if volume is the main design parameter and a slight increase in weight is not critical.

All these conclusions were further corroborated by a work authored by Sergi et al. [60], which focused on the comparison of the multiple-impact response of a 250 kg/m^3^ agglomerated cork and a 130 kg/m^3^ PVC foam. For a seven J-impact, agglomerated cork withstood 10 impacts with a permanent deformation of only 15% and a logarithmic increase in the maximum force, whereas the PVC foams showed a permanent deformation of 57.2% and a linear increase in the maximum force after only five impacts. The situation turned out to be even more severe for 10 J impacts where the agglomerated cork experienced a permanent deformation of 23% and always a logarithmic increase in the force after 10 impacts, whereas the PVC foam showed a permanent deformation of 54.4% after only three impacts. Even in this case, if a reasonable increase in the component weight can be tolerated, the application of agglomerated cork as the core material in a sandwich structure can ensure a higher dimensional stability and hence a more constant spacing of the skins throughout time, thus preserving the flexural performance of the panels that strongly depend on skins distance.

**Figure 8:**
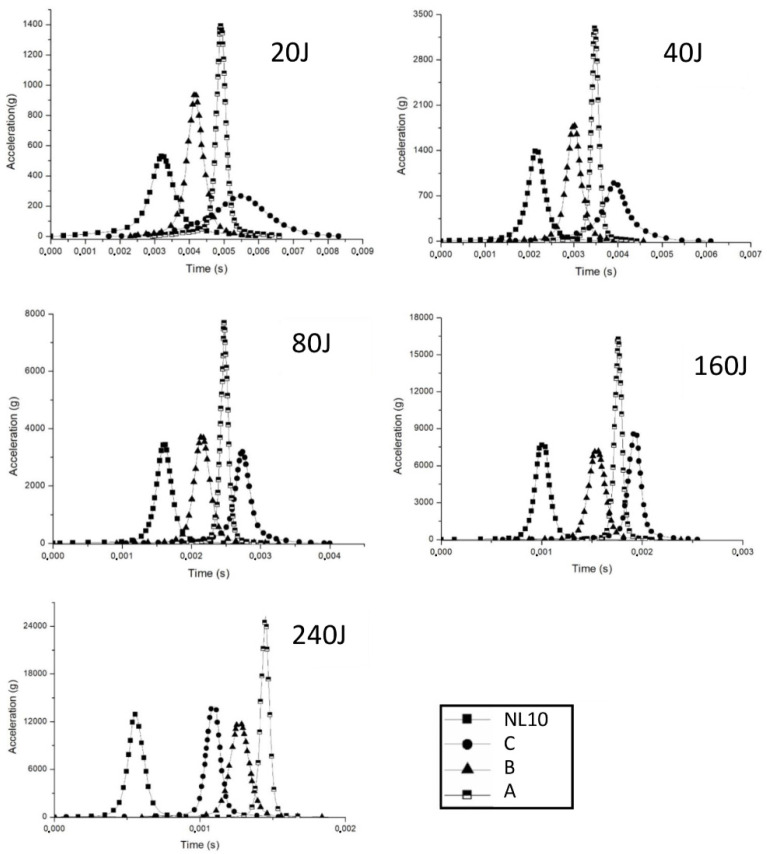
Acceleration impact test results for headbands produced with three commercial solutions A (64.90 kg/m^3^), B (309 kg/m^3^), C (218 kg/m^3^) and an agglomerated cork of 140 kg/m^3^ density (Reprinted with permission from [66]).

Due to the promising results obtained, many research studies decided to probe the real feasibility of agglomerated cork in energy absorbing devices evaluating its performance when employed as a helmet liner [66,67,68] or as car padding [69,70,71]. In their work on sport headbands, Varela et al. [66] confirmed that 140 kg/m^3^ agglomerated cork can provide the same or higher head deceleration than other commercial headbands, as proven by the numerical results shown in Figure 8 for different impact energies.

**Figure 9:**
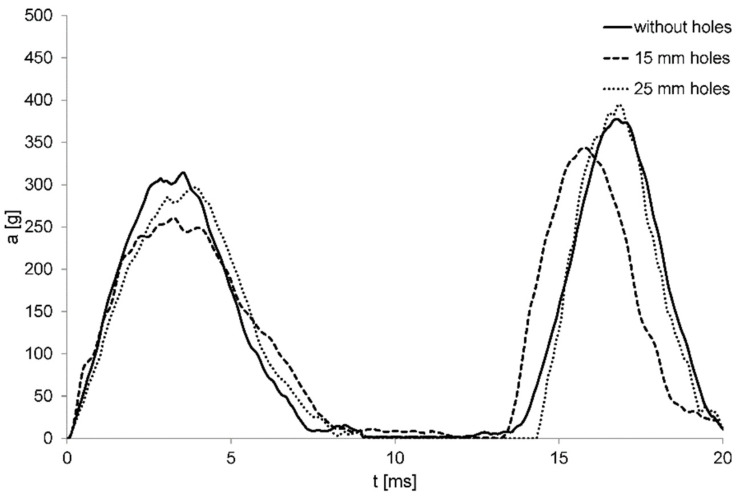
Acceleration response of the bulk and holed versions of agglomerated cork liners (Reprinted with permission from [67]).

In this specific application, agglomerated cork is absolutely competitive with commercial solution without producing a weight increase and providing an eco-friendlier piece of sport equipment. Appealing results were also presented by Fernandes et al. [67], who numerically compared the multiple-impact performance of a 40-millimeter thick helmet liner made with 90 kg/m^3^ EPS foam and 216 kg/m^3^ agglomerated cork. After the first impact, agglomerated cork and EPS foam display comparable acceleration peaks, i.e., 314.1 g and 328.3 g, respectively, but agglomerated cork provides a lower acceleration peak after the second impact, in particular a 377.7-gram acceleration against the 438.1-gram acceleration of the EPS foam. Moreover, the modification of agglomerated cork liners with the addition of 15-millimeter diameter holes allowed the liner performance to be further improve by reducing its overall weight and also the first and second peak acceleration to 260.8 g and 343.6 g, respectively, as shown in Figure 9.

Tay et al. [71] and Paulino and Texeira [70] investigated the feasibility of agglomerated cork as cellular padding to be inserted in car doors to improve the safety in case of side-impact crashes. Tay et al. pointed out that compared to other lighter polymeric foams, such as polyurethane (PUF), IMPAXX, DAX 55 and CONFOR green foams, agglomerated cork displays the worst performance from the acceleration and energy absorption point of view, providing a reduction of 30% in impact acceleration with respect to the without-padding configuration, but it is the best padding to reduce the intrusion of the impacting element in the car cabin. More promising are the results by Paulino and Texeira, who compared the performance of agglomerated cork with a PUF, an IMPAXX and an aluminum foam. According to the energy performance index proposed by the two authors to quantify the energy absorption rate, agglomerated cork is the best polymeric padding and can considerably improve car occupant safety in side-impact crashes by reducing the maximum acceleration value by 54%, increasing the energy absorbed by the structure by 9% and reducing the maximum intrusion by 10%.

## 4. Conclusions

The increasing demand for more eco-sustainable materials turned the attention to bio-based materials from renewable resources. If vegetable fibers are the rational solution to improve polymer composite eco-friendliness by decreasing the environmental impact of their manufacturing process and easing their disposal, other interesting solutions to improve the green side of other industrial products can be obtained by exploiting other natural materials, such as cork. For many years, this cellular material held the place of honor in wine stoppers’ production thanks to the combination of lightness, antimicrobial and good frictional properties and excellent dimensional recovery. In light of this massive exploitation, many researchers became interested in its properties and thanks to its chemical composition and microstructure, its peculiar compressive response became more and more attractive. Since 1980, many studies have investigated its anisotropic behavior and its pronounced viscoelasticity, paying particular attention to its main features: the near zero Poisson’s ratio and the exceptional dimensional recovery.

All the outcomes obtained disclosed the great potential of this material and its suitability for other industrial applications, such as an acoustic and thermal insulator in buildings and floor paneling, encouraging the optimization of cork harvesting by exploiting wine stoppers’ production by-products for the manufacturing of agglomerated cork planks. This natural multi-scale foam not only includes the peculiar features that characterize natural cork but can also tailor its mechanical properties by changing the polymeric binder type and concentration, granule size and plank density to address specific application requirements.

The good energy absorbing capabilities, due to its cellular structure and viscoelastic nature, and the unique dimensional recovery make agglomerated cork a good alternative to the common synthetic foams employed as core material. Indeed, it allows sandwich structures’ impact resistance and damage tolerance to be improved and their dimensional stability to be increased, which is fundamental to ensure a constant distancing of the load-bearing skins and hence of the flexural properties of the structure. Moreover, the aforementioned properties provide the agglomerated cork with a good crashworthiness, which makes it a promising candidate in safety and energy absorbing devices, especially when a multiple-impact resistance is required. With respect to other traditional foams, agglomerated cork is able to reduce the acceleration peaks resulting from consecutive impacts, keeping its dimension and its energy absorbing capabilities almost unchanged. This makes it a realistic bio-based solution to be applied in the production of sport headbands and helmets or as car doors padding, as already proven by some preliminary research studies.

## 5. Future Perspectives

From the crashworthiness point of view, the next step would lie in the production of real prototypes to verify the compliance with the safety requirements established by the regulations such as the ECE R22.05, which is the European helmet safety standard responsible for the certification of motorcycle helmets and which is also recognized outside the European Union. The verification of the fundamental requirements may encourage the application of this material at an industrial scale leaning on its eco-sustainable nature and wiping out manufacturers’ diffidence.

To be applied as core material, many other points need to be addressed. A high out-of-plane stiffness is one of the main requirements for a valid core material. Due to its soft nature, agglomerated cork is able to provide an improved dimensional stability and impact resistance to the sandwich structure, but the resulting flexural properties are lower than the ones achievable with other core materials. In this regard, an investigation of different polymeric binders able to improve agglomerated cork stiffness without jeopardizing its good damping capabilities is needed. Some attempts were proposed by Castro et al. [72] and Silva et al. [73], who investigated the feasibility of novel agglomerated cork produced with an epoxy resin, disclosing significant improvements in the quasi-static and impact properties of the overall sandwich structure. In this line, the identification of a high-performing and more eco-friendly resin would allow the green profile of the core material to be improved [74].

Moreover, the application of agglomerated cork as core material may imply its exposure to harsh environments, thus requiring an investigation of the material compressive response evolution as a consequence of this exposure. For example, a marine environment entails the combined presence of water, salts, direct sunlight, wind, etc., whereas the winter period may require the investigation of freeze–thaw cycles, which may affect negatively cork’s microstructure. In this regard, a recent study by Sergi et al. [75] addressed the effect of sea water and salt fog exposure on the compressive behavior of agglomerated cork, assessing how the stability of this material is affected by this type of environment. Even in this case, a strong plasticizing effect of water and salt fog on agglomerated cork’s compressive response was disclosed together with a partial recovery of the compressive performance as a consequence of drying. It was also proven that the presence of salts deriving from sea water plays a healing effect, determining a better recovery of the mechanical properties with respect to pure water. In light of these results, the durability of cork and cork-related products is a mandatory item in future research, especially considering new intended applications in the automotive industry as seals and gaskets, in aerospace as thermal protection systems for next generation space exploration missions or as a filler between expansion joints in concrete slabs in the construction field.

## Figures and Tables

**Table 1:** General properties of cork.

Property	Value	Ref.
Density [kg/m^3^]	120–240	[10]
	200–400	[11]
Electrical Conductivity [S/m]	1.26 × 10^−10^ (25 °C) 1.67 × 10^−13^ (50 °C)	[12]
Acoustic Resistivity [kg/(m^2^ s)]	1.2 × 10^5^	[13]
Thermal Conductivity [(W/(m K))]	0.045	[14,15]
Specific Heat [J/(kg K)]	350	[14]
Permeability [mol/(m s Pa)]	280.5 × 10^−13^ (Liquid water) 110.1 × 10^−13^ (Water Vapor)	[16]
Friction Coefficient	0.97 (radial) 0.77 (non-radial)	[17]

**Table 2:** Chemical composition of cork.

Component	Pereira [22]	Gil [14]	Pereira [23]			
Suberin	42.8%	42%	37.8%	40.3%	35.2%	41.2%
Lignin	22%	21.5%	21.7%	22%	22.4%	20.7%
Polysaccharide (Cellulose/Hemicellulose)	19%	16%	18.5%	15.7%	21.3%	17.2%
Extractives (Water, Ethanol, Dichloromethane)	16.2%	13%	15.7%	14.3%	16.9%	14.1%
Others	-	7%	-	-	-	-

**Table 3:** Compressive modulus and plateau stress at 5% of strain values provided by different authors for the three main directions of natural cork.

	Compressive Modulus (MPa)			Plateau Stress (MPa)		
	Radial	Axial	Tangential	Radial	Axial	Tangential
Gibson [9]	20 ± 7	13 ± 5	13 ± 5	0.8 ± 0.2	0.7 ± 0.2	0.7 ± 0.2
Pereira [24] ^1^	11.5 ± 1.0 13.2 ± 0.7 9.9 ± 0.4	10.9 ± 0.6 12.0 ± 1.5 9.2 ± 0.5	8.6 ± 0.7 9.6 ± 1.4 7.9 ± 1.3	-	-	-
Anjos [25] ^2^	17.9 ± 2.86 18.6 ± 3.31	16.6 ± 1.76 17.1 ± 2.27	13.4 ± 1.42 11.2 ± 1.73	0.61 ± 0.057 0.59 ± 0.068	0.59 ± 0.061 0.57 ± 0.104	0.56 ± 0.044 0.44 ± 0.048
Anjos [26] ^3^	17.39 ± 4.53 22.57 ± 5.07 26.12 ± 4.5	16.61 ± 3.28 16.26 ± 2.57 18.53 ± 5.19	14.39 ± 4.79 16.75 ± 3.88 19.07 ± 4.47	0.72 ± 0.09 0.74 ± 0.15 0.74 ± 0.11	0.57 ± 0.08 0.32 ± 0.14 0.52 ± 0.11	0.65 ± 0.10 0.56 ± 0.21 0.75 ± 0.09
Oliveira [27] ^4^	10.4 ± 3	9.2 ± 2.6	9.2 ± 2.6	-	-	-

^1^ Values for three corks with different calibers, i.e., small (2 mm), medium (3.5 mm) and large (6 mm); ^2^ Values for two different commercial qualities of cork, i.e., class 1 good and class 4 poor; ^3^ Values for three corks with different density, i.e., 0.11–0.15 g/cm^3^, 0.15–0.19 g/cm^3^ and 0.19–0.25 g/cm^3^; ^4^ Mean values obtained considering cork from 10 different production sites; plateau stress is not reported because it was calculated at 20 and 30% strain.

**Table 4:** Natural cork Poisson’s ratios.

	Poisson’s Ratio		
	R/NR	NR/R	NR/NR
Gibson et al. [9]	0 ± 0.05	0 ± 0.05	0.5 ± 0.05
Fortes et al. [37]	0.097	0.064	0.26

**Table 5:** Effect of an increase in the design parameters under consideration on the compressive stiffness of agglomerated cork.

	Compressive Modulus (MPa)		
	Santos [41]	Crouvisier-Urion [42]	Jardin [43]
Binder type (Rigidity)	↑	↑	none
Binder concentration	=↓	=	none
Grain Size	↑	↑	↑
Density	↑	none	↑

**Table 6:** Effect of an increase in the test parameters under consideration on the compressive stiffness of agglomerated cork.

	Compressive Modulus (MPa)	
Out-of-plane	↓	[47,48]
Strain rate	↑	[47,48,49,50,51,52,53]
Moisture	↓	[42]
Temperature	↓	[49,50]

## Data Availability

Data sharing not applicable.
